# Integration of Dietary Fibre for Health Benefits, Improved Structure, and Nutritional Value of Meat Products and Plant-Based Meat Alternatives

**DOI:** 10.3390/foods14122090

**Published:** 2025-06-13

**Authors:** Nikola Stanišić, Vladimir S. Kurćubić, Slaviša B. Stajić, Ivana D. Tomasevic, Igor Tomasevic

**Affiliations:** 1 Institute for Animal Husbandry Belgrade—Zemun, Autoput Beograd-Zagreb 16, 11000 Belgrade, Serbia; nikola0135@yahoo.com; 2 Department of Food Technology, Faculty of Agronomy, University of Kragujevac, Cara Dušana 34, 32102 Čačak, Serbia; 3 Département of Animal Source Food Technology, Faculty of Agriculture, University of Belgrade, Nemanjina 6, 11080 Belgrade, Serbia; stajic@agrif.bg.ac.rs; 4 Institute of Meat Hygiene and Technology, Kaćanskog 13, 11000 Belgrade, Serbia; ivana.tomasevic@inmes.rs; 5 DIL German Institute of Food Technology, Prof.-von-Klitzing-Str. 7, D-49610 Quakenbrück, Germany

**Keywords:** dietary fibres, meat products, plant-based meat, texture modification, health benefits, water-holding capacity

## Abstract

This review highlights the latest research on dietary fibre (DF) applications in meat and meat analogues, providing insights into their role in shaping future food innovations. DFs provide significant long-term health benefits, such as better gut health, lower cholesterol levels, and possible protection from metabolic diseases. They also enhance the texture, juiciness, and overall quality of plant-based meat alternatives (PMAs) and traditional meat products. Among the most effective fibres, cereal-derived fibres, fruit- and vegetable-derived fibres, and legume-based fibres have been shown to improve water-holding capacity (WHC) and emulsification properties, enhancing mouthfeel and juiciness. New processing methods, such as enzymatic hydrolysis and extrusion, can change how fibres work. By combining various fibre sources with innovative processing methods, the food industry can create meat and PMA products that are not only healthier but also tastier and more sustainable.

## 1. Introduction

Dietary fibres (DFs) are used in the formulation of conventional meat products, such as emulsion-type sausages, dry-fermented sausages, and pâtés. Their incorporation can enhance texture, water retention, and emulsification stability, improving mouthfeel and extending shelf life [1]. Additionally, fibres act as fat replacers, reducing caloric content while maintaining sensory attributes [2]. Beyond their functional properties, fibre-rich ingredients enhance the nutritional profile of meat products by increasing DF intake and essential nutrients like potassium and magnesium [3]. These benefits align with growing consumer demand for healthier and functionally improved meat products.

However, increasing consumer awareness of the health risks associated with excessive consumption of red and processed meats, including colorectal cancer and other diseases linked to lipid oxidation and the formation of harmful compounds during high-temperature cooking, has driven demand for alternative protein sources [4]. In response, the market for plant-based meat alternatives (PMAs) is expanding rapidly, with global projections estimating growth from $11.3 billion in 2023 to $35.9 billion by 2033 [5]. Despite this expansion, both conventional meat products and PMAs face challenges in achieving optimal texture, juiciness, and overall nutritional quality. One promising solution to these challenges is the strategic incorporation of DFs, which offer not only functional and health benefits but also contribute to more sustainable food production [1].

Unlike plant-based products, conventional meat naturally lacks carbohydrates. However, ingredients such as starches and fibres are frequently added to enhance functionality, structure, and nutritional value [6]. Polysaccharides commonly used in PMA production include starches, flours, and various non-starch hydrocolloids such as methylcellulose, carrageenan, xanthan, and guar gum [7]. These ingredients enhance texture, binding properties, mouthfeel, and juiciness by retaining water within the product. Interestingly, the carbohydrates used in plant-based meat production largely overlap with those used in traditional meat products, with methylcellulose as one notable exception [8]. This modified cellulose DF, commonly used as a heat-activated binder in PMAs, is rarely found in conventional meat products [9]. Given the industry’s push towards E-number-free formulations, manufacturers are seeking alternative binding agents.

DFs naturally occur in various plant sources, including cereals, fruits, vegetables, and legumes. Additionally, fibre-rich industrial by-products, such as apple pomace, citrus peel, grape skin, and sugar beet pulp, contain valuable fibre fractions like cellulose, lignin, and pectin [10,11]. Incorporating these by-products into meat and PMAs not only enhances nutritional quality but also promotes a circular economy by reducing food waste and improving sustainability [12].

From a functional perspective, DFs contribute to texture, water-holding capacity (WHC), and emulsification, while also providing health benefits such as cholesterol reduction and improved digestion [13]. In processed meats, fibres help mitigate lipid oxidation and serve as fat replacers, making them valuable components of functional meat products [2]. In PMAs, one of the biggest challenges is replicating the fibrous texture of animal muscle, contributing to the chewiness and overall mouthfeel of meat. While plant proteins such as soy, pea, and wheat provide some structural integrity, they often fail to fully mimic the fibrous properties of real meat [14]. To address this, advanced food processing techniques such as extrusion, shear cell processing, and 3D printing, are being explored to improve texture. DFs are now recognised as key ingredients that enhance binding properties and moisture retention in these formulations.

Despite these advantages, incorporating DFs into meat and PMAs requires careful formulation to maintain desirable sensory attributes. Different fibre sources—cereal-based (wheat, oat, rice bran), fruit-derived (apple, citrus, grape pomace), legume-based (pea, soy, lentil), and seaweed-derived (agar, alginate, carrageenan)—offer unique functional properties that must be tailored to specific applications [3]. Additionally, emerging fibre modification techniques, such as enzymatic hydrolysis, ultrasonication, and extrusion processing, are being studied to enhance fibre functionality in food systems [15]. These modification techniques lead to changes in the structure, physicochemical properties, and biological activities of the fibre. The changes are related to particle size, crystal structure, water-holding properties and swelling, oil emulsion properties, and antioxidant activity as well [15]. Therefore, insoluble DFs can be modified according to their application in different food systems.

This review explores recent advancements in DF applications for processed meat and PMAs. By highlighting their role in improving texture, water retention, and overall product quality, we aim to support the development of healthier, more sustainable meat and plant-based alternatives that align with consumer expectations and industry needs.

## 2. Nutritional and Health Benefits

The incorporation of DFs offers a wide range of benefits that not only enhance the nutritional profile of meat and PMA products but also contribute to improved health of final consumers. As globalization continues to shape dietary habits, there is a growing demand for healthier food options, particularly in light of rising global health concerns such as obesity, cardiovascular diseases, and diabetes, which are often linked to low fibre intake [16,17]. By integrating DFs from sources like legumes, cereals, and seaweeds, manufacturers can improve the functional properties of meat products while promoting better health outcomes [1,18]. This shift has the potential to transform processed meats from being perceived as high-fat, unhealthy options to more balanced, nutrient-rich alternatives that align with modern dietary guidelines [19].

Epidemiological studies indicate that the consumption of foods containing DF helps to reduce the occurrence of obesity, some types of cancer, CVD, and gastrointestinal diseases [20]. A daily intake of 25 to 30 g of DF is considered optimal, according to the recommendations of the American Heart Association [21]. Non-communicable diseases (NCDs), which include CVD, cancer, and type 2 diabetes, represent current public health issues and are the primary causes of death (about 70% of mortality worldwide). Prevention measures [22,23,24,25], especially those involving high-quality diets that include functional foods or ingredients, are increasingly emphasised [26]. Epidemiological studies reveal a positive correlation between a diet rich in polyphenols and a reduction in the risk of CVD and certain types of cancer [27]. A main characteristic of commercial grape fibre is its high content of total dietary fibre (TDF), protein (8.6–10.8%), minerals (1.3–3.8%), and fat (2.8–8.6%). This fact opens up the possibility of using initial by-products as ingredients in the food industry, as they have a high TDF content [28].

The Food and Drug Administration (FDA) of the United States, Department of Health and Human Services has accepted two major health claims for DF. The first confirms that it is possible to reduce the incidence of several types of cancer with reduced dietary fat intake and high consumption of DF (derived from various sources such as fruits, vegetables and/or cereals). Another FDA claim confirms that diets low in cholesterol and saturated fat and high in fruits, vegetables, and whole grains reduce the risk of cardiovascular disease (CVD) [29].

### 2.1. Prevention and Control of CVD

Continuous intake of the recommended level of DF lowers blood cholesterol levels, thereby reducing the risk of death from stroke and CVD [30]. The potential to preserve and improve heart health through diet is realised by understanding the biochemical and molecular pathways. Increasing the level of high-density lipoprotein (HDL) in the blood can be achieved by enriching the diet with oatmeal, nuts, fruits, citrus fruits, pears, etc., which leads to the removal of stiffness of blood vessels and the removal of deposited low-density lipids (LDL) [31]. LDL and triacylglycerols usually act as the main molecules for clogging the blood flow through the arteries. LDL from the arteries returns to the liver where it is metabolised under the influence of HDL, which removes it from the blood [32]. Cholesterol-induced blockages of the coronary arteries and heart attacks are prevented in this way [29]. High DF intake maintains arterial pressure within physiological limits, thereby reducing the potential for heart attack [33]. Increased arterial stiffness is another consequence of high HDL levels, and indirectly leads to a decrease in insulin resistance in humans [31,34].

Some specific DFs (guar gum, β-glucan, psyllium, and pectin) increase bile acid excretion in the faeces [35] by inhibiting their reabsorption in the body [36]. Cholesterol is converted to bile acids, which, together with bile and phospholipids, form mixed micelles that subsequently solubilize cholesterol. In this way, bile acids are utilised and are not available for reabsorption. Bile acid synthesis is possible due to the circulation of cholesterol in the body, and bile acids indirectly reduce the cholesterol titer in the blood [37].

Of particular importance is the fact that there is a positive correlation between high intake of DFs and a reduction in premature mortality rates in patients with existing CVD and hypertension, thought to be due to a reduction in total cholesterol and low-density lipoprotein cholesterol levels, as well as a reduction in systolic and diastolic blood pressure [38].

It is believed that fermentation of DFs produces short-chain fatty acids that may mediate the hypocholesterolemic effects of DFs. Propionate facilitates the reduction of blood cholesterol levels by inhibiting cholesterol synthesis in liver cells. The viscosity of soluble fibre may prevent cholesterol absorption, and glucose and fibre viscosity improve glycemic control and cholesterol concentration.

### 2.2. Obesity Prevention with DF-Rich Meals

The feeling of satiety and fullness occurs due to the lower energy density and high fibre content of DF-rich meals. The consumption of such meals reduces the volume of the remaining meal and controls food intake, thus preventing the development of obesity in humans [39]. Higher DF intake controls blood sugar levels because tissues do not absorb or break down fibre; blood sugar levels do not increase as when consuming a carbohydrate diet. A diet rich in DFs modulates insulin resistance and maintains blood sugar levels within the permissible range.

This mechanism controls body fat storage and obesity [16,40]. Higher intake of DFs reduces insulin secretion, which mobilizes fat from depots [41]. The rate of body weight loss is directly proportional to the level of DF consumption [42] and their type [39], as they facilitate rapid gastric emptying by reducing the transit time of digested food in the gastrointestinal tract, and consequently reduce food absorption in the body.

### 2.3. Diabetes Control: The Impact of a Diet High in DFs

A diet low in fibre and high in glycemic index is associated with the development of type 2 diabetes in humans [43]. DFs from some whole grain foods reduce postprandial blood glucose levels, which reduces insulin requirements, thereby preventing pancreatic overload and controlling diabetes [44]. Due to their high WHC, ingested DFs interfere with gastric emptying by forming a gel matrix [45], which can thicken intestinal contents and reduce the interaction between food and digestive enzymes. This results in slower digestion and absorption of carbohydrates [46], which further contributes to diabetes control.

### 2.4. Cancer Prevention with Diets Rich in DFs

Dietary supplements containing functional nutraceuticals have been shown to be beneficial for human health [33,47], and DFs in particular demonstrated potential in protecting against colon, breast, and prostate cancers [48]. Data from European and American populations show that dietary DF intake reduces the risk of colon cancer. In the period between 1980 to 1981, 32 studies examined the association between DF intake and cancer, with 25 confirming an inverse relationship between DF and cancer risk [49]. More recent research results published in PubMed between 1980 and 2017 were performed in a meta-analysis in 2018, the purpose of which was to find out the association between cancer risk and a diet low in DF. The results revealed a statistically significant reduction in the relative risk of colon, esophageal, gastric, and pancreatic cancers. If the recommended dietary fibre intake of 38 g/day is met, cancer mortality rates may be 17% lower [50]. Various molecular and biochemical pathways reduce cancer risk when consuming DF in the diet, as revealed by recent systematic reviews [51,52]. It is scientifically confirmed that diets rich in DF significantly contribute to the prevention and control of colon cancer, by increasing fecal mass and preventing the interaction of cancer-causing agents with the intestinal mucosa [53], or by inhibiting the production of carcinogens in the colon [54]. Moreover, SCFAs produced in the colon during fermentation of DF-rich diets play a significant role in reducing the incidence of colorectal cancer [55]. By reducing the production of secondary bile acids at acidic pH and increasing cell proliferation, butyric acid prevents the occurrence of malignancy in cells [56]. Certain DFs also act as antioxidative (AO) agents and strengthen the human immune system [57]. Results have also been published revealing the protective effect of DFs in controlling breast cancer by increasing the loss of estrogen in the fecal mass, which is a strong cause of breast cancer.

### 2.5. Antioxidative Activity

The concept of antioxidant dietary fibres (ADF), which significantly contribute to better health of the human population, implies a combined beneficial effect of both dietary fibres and antioxidants. Therefore, attention must be focused on the isolation or development of different natural AO [58,59,60].

Oxidative stress, a factor in the development of atherosclerosis, can be minimised by regularly consuming DFs containing additional antioxidants [61].

The attention directed towards the development of DFs imposes a number of questions, such as the mechanism of their action (e.g., proanthocyanidins) [62]. All research on the mechanisms of action of natural preventive agents in the living world is valuable, such as the review by Lizarragi et al. [63], which suggested that GADF induces a protective gene expression profile in the colon [64].

Consumers prefer convenience foods and foods that are low in calories, cholesterol, and fat, but also high in fibre [65]. DFs are food components composed of polysaccharides, oligosaccharides, and lignin, which are resistant to hydrolysis by enzymes in the human digestive tract [66]. Good sources of DFs are cereals, legumes, fruits, vegetables, nuts, seeds, and agro-industrial co-products [25,67,68,69,70].

The most important secondary product of winemaking is grape pomace (GP), whose imposing chemical composition is promising due to its richness in increasingly necessary DFs and compounds of exceptional AO power, such as polyphenols (anthocyanins, flavonols, flavan-3-ols, procyanidins), phenolic acids, and resveratrol. Although GP is a waste, it also serves as a raw material for obtaining distillates, extracting tartaric acid, and producing dyes of natural origin (enocyanin). More recently, GP has shown great potential for innovative applications, including the extraction of fibres enriched with bioactive ingredients, and the design of extracts with antioxidant activity [71,72,73,74,75,76,77].

The uncertainty and concern of consumers due to the use of synthetic antioxidants in food have been going on for a long time and require reliable solutions using natural alternatives from different plant sources. Research into potentially harmful antioxidants is ongoing; the issue is viewed from different angles. A recent study investigated the transfer of beta-hydroxytoluene (BHT) from packaging materials. Four synthetic antioxidants were identified in food for the first time, with BHT accounting for 76.8% and 67.6% of total BHT intake for children and adults, respectively. Therefore, food consumption is considered a significant source of BHT exposure, highlighting the need for subsequent evaluation of human exposure to these potentially carcinogenic and mutagenic substances [77,78].

### 2.6. Role in Reducing Fat and Caloric Content

DFs, especially soluble types like pectin, β-glucans, and resistant starch, are important for reducing the fat and calorie content in meat and meat alternatives. By using these fibre-rich ingredients to replace or supplement fats, manufacturers can reduce the overall fat content while keeping the texture and mouthfeel intact [79]. Soluble fibres, which are generally low in calories, function as bulking agents that add volume and weight to the final product, making them attractive to health-conscious consumers. For example, oat β-glucan is a popular ingredient known to help reduce fat absorption and enhance the texture of low-fat meat products [80,81]. Additionally, resistant starch can help increase feelings of fullness, which supports weight management efforts [82]. Overall, the inclusion of these fibres not only improves the healthiness of food products but also keeps them tasty.

### 2.7. Prebiotic Effects and Gut Health Benefits

Many DFs, including those derived from fruits, legumes, and cereal grains, possess prebiotic properties that promote gut health. Prebiotics serve as a food source for beneficial gut microbiota, such as *Bifidobacteria* and *Lactobacilli*, which are essential for maintaining a healthy digestive system [15]. The fermentation of these fibres by gut bacteria produces short-chain fatty acids (SCFAs), such as butyrate, acetate, and propionate, which support gut integrity, reduce inflammation, and enhance overall digestive health [15]. For instance, pea fibre has been shown to improve gut microbiota composition and increase SCFA production, making it a valuable ingredient in PMAs [83]. Furthermore, certain fibres, such as inulin and fructooligosaccharides, have demonstrated potential in alleviating symptoms of digestive disorders like irritable bowel syndrome and in improving immune function [79].

### 2.8. Potential Reduction of Process-Induced Toxicants

DFs from plant sources such as grape pomace, wheat bran, and oats have been shown to possess AO properties that help mitigate the formation of harmful process-induced toxicants in food products. During the production of meat and PMA products, high processing temperatures and chemical reactions can lead to the formation of toxic compounds, including advanced glycation end-products (AGEs) and polycyclic aromatic hydrocarbons (PAHs), which are associated with chronic diseases such as cardiovascular diseases and cancer [84,85]. The antioxidant and chelating properties of fibres can help reduce the formation of these harmful compounds, improving the safety and nutritional profile of the final product. For example, grape pomace fibre has been shown to reduce oxidative stress and inhibit the formation of AGEs during food processing [11]. Similarly, wheat bran fibre has demonstrated the potential to reduce PAH levels in grilled meat products, offering the dual benefit of enhanced safety and improved texture [86].

By adding fibres that are rich in antioxidants, producers can develop products that not only meet nutritional standards but also cater to the growing consumer demand for healthier and safer food choices [8,85].

## 3. Types and Sources of Dietary Fibres Used in Meat Processing

DFs used in meat processing are derived from various plant-based materials and can be classified based on their origin and functional properties. These fibres include cereal-based fibres, legume-derived fibres, fruit and vegetable fibres, as well as novel sources such as seaweed-derived fibres and microbial-derived polysaccharides (Table 1). Each type of fibre exhibits unique physicochemical properties that contribute to specific functional attributes in meat and meat analogue products [87].

### 3.1. Cereal and Grain-Based Fibres

Cereal and grain-based fibres, including wheat, oat, barley, and rice bran fibres, are among the most widely used fibres in the food industry. These fibres are rich in insoluble polysaccharides such as cellulose, hemicellulose, and lignin, which contribute to improved WHC, fat absorption, and textural enhancement in meat formulations [13]. Wheat bran is particularly high in arabinoxylans, which have prebiotic effects and may enhance gut microbiota diversity [88]. Oat fibres, including β-glucans, are notable for their cholesterol-lowering effects and ability to modulate blood glucose levels [80,89 ]. Barley β-glucans share similar properties and have been associated with reduced cardiovascular disease risk [90]. Rice bran fibre, rich in ferulic acid and other antioxidants, has gained attention for its potential role in reducing oxidative stress and inflammation [91].

### 3.2. Legume-Derived Fibres

Legume-derived fibres, such as pea, soy, and lentil fibres, have gained popularity due to their structure and excellent WHC. Pea fibre has been reported to improve the structural integrity of plant-based meat analogues by enhancing their fibrous texture and elasticity [83]. Soy fibre, derived from soybean hulls, is composed of hemicellulose, cellulose, and pectin, and has been linked to improved bowel function and reduced cholesterol absorption [92]. Moreover, legume fibres contain bioactive compounds such as isoflavones, which provide antioxidant properties that can enhance the shelf life of meat products [19]. Recent studies have also highlighted the potential of lentil fibre, rich in resistant starch and oligosaccharides, to improve the texture and nutritional value of meat products, particularly in low-fat formulations [2].

### 3.3. Fruit and Vegetable Fibres

DFs derived from fruits and vegetables, such as apple, citrus, and carrot, are increasingly utilised in meat formulations due to their high pectin content and beneficial gelling properties [93]. Apple fibre, primarily composed of pectin and cellulose, has been demonstrated to enhance the texture and yield of low-fat sausages while improving dietary fibre intake [94]. Citrus fibre, derived from orange and lemon peels, is high in pectin and flavonoids and is often added to meat formulations to enhance juiciness and reduce syneresis, particularly in cooked and frozen products [95]. Carrot fibre, containing both insoluble cellulose and soluble pectin, has shown promise as a stabilizing agent in emulsified meat systems, improving WHC and protein interactions [86]. Additionally, fibres from other sources, such as beetroot and tomato, have been explored for their potential to improve the colour and sensory properties of both meat and PBM products [8,79].

### 3.4. Seaweed and Microbial-Derived Fibres

Due to their superior gelling and emulsifying properties, seaweed-derived fibres, such as alginate and carrageenan, have been widely employed in meat processing [18]. Alginate, found in brown algae, is frequently used in restructured meat products to improve binding properties, whereas carrageenan, extracted from red seaweed, is commonly included in processed meats to enhance water retention and reduce fat separation [30]. In recent years, microbial-derived polysaccharides such as xanthan gum and bacterial cellulose have been investigated for their potential to improve the structural and textural properties of plant-based meat analogues [96]. Microbial-derived polysaccharides, such as xanthan gum, have been shown to enhance the viscosity and stability of meat emulsions [97]. Furthermore, bacterial cellulose has gained attention for its unique structural properties and potential applications in functional foods [98].

## 4. Functional Roles of Fibres in Meat and Plant-Based Meat Products

DFs play an important role in the physical, chemical, and sensory properties of both traditional meat products and plant-based meat alternatives. Manufacturers can enhance WHC, replace fat, stabilize emulsions, and improve texture by incorporating fibres into meat formulations (Table 1). These functional benefits make DFs indispensable in creating healthier, lower-fat, and fibre-enriched meat products [13,16,87].

**Table 1:** Commonly used DFs and their roles in meat and PMA products.

Dietary Fibre	Functional Role	Potential Health Benefits	Product Type	Concentration Range (*w*/*w*)	References
Oat β-glucan	Improves emulsion stability, reduces fat absorption, enhances texture, and increases water retention.	Lowers cholesterol levels, improves satiety, and supports gut health.	Low-fat sausages, burgers	1–5%	[80,89]
Pea fibre	Enhances fibrous texture, improves structural integrity, and increases WHC.	Promotes gut health, increases SCFA production, and supports weight management.	Plant-based meat analogues	2–10%	[9,83]
Wheat fibre	Improves WHC, reduces cooking loss, and enhances texture.	Supports digestive health, reduces caloric density, and improves satiety.	Sausages, meatballs	1–7%	[99]
Citrus fibre	Decreases residual nitrite levels and favours micrococcus growth.	Decreases the risk of nitrosamine formation	Fermented sausages	1–2%	[79]
Apple fibre	Improves texture, increases yield, and enhances water retention.	Increases DFs intake, supports gut health, and provides antioxidant benefits.	Low-fat sausages, burgers	1–5%	[91]
Carrageenan (seaweed)	Enhances water retention, reduces fat separation, and stabilises emulsions.	Supports gut health, improves texture, and provides antioxidant properties.	Processed meats (e.g., ham)	0.1–1%	[1]
Resistant starch	Acts as a bulking agent, reduces caloric density, and improves texture.	Promotes gut health, increases SCFA production, and supports weight management.	Low-fat meat products	2–8%	[82]
Rice bran fibre	Improves water retention, enhances texture, and provides antioxidant properties.	Reduces oxidative stress, supports gut health, and lowers cholesterol levels.	Meat patties, sausages	1–5%	[3,91]
Grape pomace fibre	Reduces oxidative stress, improves texture, and enhances water retention.	Provides antioxidant properties, reduces AGEs and PAHs, and supports cardiovascular health.	Meat analogues, sausages	1–4%	[11,85]
Potato fibre	Enhances firmness, improves WHC capacity, and increases yield.	Supports gut health, improves satiety, and provides antioxidant properties.	Meat burgers, sausages	2–6%	[100]
Four various proportions of buckwheat husk (BH)	Positive effect on water retention during the storage of frankfurter-type sausages; significant effect on the amino acid content.	Good dietary source of essential amino acids, trace elements and phenolic compounds; preserved protein digestibility; significant effect on mineral content.	Frankfurter-type sausages	0% BH = 0 g, 1% BH = 4 g, 2% BH = 8 g and 3% BH = 12 g	[101]
Chia (*Salvia hispanica* L.) mucilage powder	Fat substituent	Substitute saturated fat (SFA); improved technological characteristics and additional healthier claims.	Emulsified meat products	2.5% and 5.0% (*w*/*w*)	[102]
Oat bran powder	Improving physico-chemical quality	Crude fibre content significantly higher in all treatment groups; ash content increased significantly	Emulsion-type pork sausages	3.0%, 6.0%, and 9.0% (*w*/*w*)	[103]
Orange fibre, wheat fibre, bamboo fibre, carrot fibre	Quality maintenance during storage	All the fibres could prevent the growth of spoilage bacteria	Emulsion-type chicken sausage	1.0% (*w*/*w*)	[104]
Orange fibre, wheat fibre, bamboo fibre, carrot fibre	Development of functional meat products	All of the fibres could prevent the progress of oxidation	Mortadella	1.0% (*w*/*w*)	[105]
Lyophilised vegetables	Phosphate substitutes	Progress towards the “clean-label” concept	Sausage meat	1.0%, 1.6%, 2.2%, 2.8%, 3.4%, and 4.0% (*w*/*w*)	[106]
Guava and tomato waste powders (peels and seeds)	Enhance and improve shelf-life	Good source of DFs and bioactive compounds	Beef burger	5.0%, 10.0%, and 15.0% (*w*/*w*)	[107]
Apple pomace (rehydrated, from dried powder)	Enhancing nutritional and antioxidant properties	The improved fibre and phenol content, lower fat and calories	Italian salami	7.0% and 14.0% (*w*/*w*)	[108]
Inulin, chitosan, carboxymethyl cellulose, pectin, cellulose	Fibre enrichment/fat substitutes	Changes in water distribution caused by heating are a consequence of the enrichment with chitosan.	Comminuted meat products	2.0% (*w*/*w*)	[109]
Red lentil	Quality enhancement and creation of the low-fat product	Increased the moisture and protein content in beef burger	Beef burger	5.0% and 10.0% (*w*/*w*)	[110]

### 4.1. Water-Holding and Fat-Binding Capacity

Water retention is a key factor in meat products, directly impacting yield, juiciness, and texture. DFs are excellent at binding water thanks to their porous structure and unique chemical composition. For example, Stanišić et al. [100] found that potato fibre outperforms wheat and oat fibres in WHC, retaining up to 9.5 g of water per gram of fibre. This makes it an excellent functional ingredient for improving moisture retention in meat products. Similarly, wheat and oat fibres have been shown to reduce cooking losses in low-fat meat products, enhancing juiciness and tenderness [81,92]. Pea fibre has also demonstrated significant water-binding properties, making it a valuable ingredient in traditional and PMA products [82].

Fibres also bind fat, which is crucial for maintaining texture and stability in meat products. Fibres can prevent fats and oils from leaking out during cooking, which improves the mouthfeel, especially in low-fat options [1]. Specific fibres, such as oat β-glucan and resistant starch, interact with lipids to reduce fat absorption and enhance emulsion stability, further contributing to the quality of healthier meat products [80,89]. Recent studies have also highlighted the potential of rice bran fibre to improve fat-binding capacity while adding antioxidant benefits to meat formulations [91].

### 4.2. Emulsification and Gelling Properties

Emulsification is particularly important in processed meats like sausages and restructured products. Fibres help stabilize emulsions by binding water and fat, forming a gel-like network that prevents separation [95,97]. As stated by García et al. [94] and Fernández-López et al. [79], citrus and apple fibres improve the stability of meat emulsions, ensuring a consistent texture and preventing phase separation. Seaweed-derived fibres like alginate and carrageenan are also widely used in processed meats for their exceptional emulsifying and gelling properties, which enhance product stability and texture [18,111]. Furthermore, as a microbial-derived polysaccharide, xanthan gum has been shown to improve the viscosity and stability of meat emulsions, making them commonly used in the meat processing industry [98].

In PMAs, fibres also serve as structuring agents, helping to replicate the fibrous texture of real meat. Pea and soy fibres, for example, are particularly effective at creating a meat-like texture in PMAs, improving their structural integrity and making them more appealing to consumers [83,96]. These fibres enhance texture and contribute to the overall sensory experience, bringing PMAs closer to traditional meat products [86].

### 4.3. Texture and Sensory Enhancement

Texture is one of the most important factors influencing the overall sensory quality of food. The addition of DFs can modify textural attributes like hardness, chewiness, and cohesiveness. For example, Stanišić et al. [100] reported that potato and oat fibres significantly increase firmness when added to meat burgers, improving their texture without sacrificing sensory scores. Similarly, wheat fibre has been shown to enhance the mouthfeel of low-fat sausages by boosting juiciness and cohesiveness [81,92]. Additionally, carrot fibre has been found to improve the texture of emulsified meat systems by enhancing WHC and protein interactions [86].

Fibres also influence sensory characteristics like colour and flavour. While some fibres, such as citrus and apple, enhance juiciness and texture, others may introduce off-flavours if not carefully selected [79,93,]. Choosing the right fibre type and concentration to achieve the desired functional benefits without compromising sensory quality is crucial in the meat and PMA processing industry. For instance, beetroot and tomato fibres have been used to improve the colour and sensory properties of PMA products, making them more appealing than the original meat products [,^79^].

In summary, DFs have various functional effects when incorporated into meat and PMA products. Moving forward, research should focus on optimizing the use of fibres to maximize their functional advantages while ensuring minimal impact on sensory quality and consumer acceptance.

### 4.4. Fibres in Fat-Reduced and/or Fat-Improved Meat Products

DFs were not only used in “regular” meat products to improve technological and sensory properties [112,113] but were also used in the creation of meat products with improved nutritional quality (Table 2) and as additive replacements. Due to their previously mentioned properties (water retention, emulsification properties, etc.), DFs have been used in reduced-fat meat products and/or in oil systems that partially (or totally) replace fatty tissue [114,115,116]. Moreover, good emulsification properties enable their potential use as phosphate replacers in emulsion-type sausages, with the aim of creating “clean label” products [117,118].

Fat plays an essential role in the overall quality of meat products—it participates in the creation and development of desirable technological properties such as processing yield, continuous and uniform drying (in dry-fermented sausages), formation and stabilization of meat emulsions (in emulsion-type sausages), sliceability, colour, texture, etc. [99,123,]. Moreover, it is also important for the development of sensory properties—odour, taste, juiciness, mouthfeel, and hardness [124]. From a nutritional point of view, fat is a source of energy, essential fatty acids, and a carrier of fat-soluble vitamins in meat products [109]. On the other side, high energy intake from animal fat, as well as its high content of saturated fatty acids (of animal fat), is associated with a higher risk of cardiovascular diseases, diabetes, etc., which has driven demand for healthier meat products. However, bearing in mind the above, developing meat products with reduced fat content poses quite a challenge.

Fat reduction is usually based on two main criteria: the reduction of fat by increasing the content of lean meat, and the reduction of fat by replacing it with non-lipid fat replacers (e.g., plant proteins, hydrocolloids, DFs) that have little or no caloric content [109,125]. The first approach has limitations, as decreasing fat content in the product formulation can significantly interrupt the production process (e.g., drying of fermented sausages) and reduce acceptability [109,126].

On the other hand, the use of non-lipid fat replacers such as various DFs can resolve this. DFs have been successfully used as fat replacers in different grounded meat products, namely fermented sausages, emulsion-type sausages, and burger-type meat products.

Fat replacement in fermented sausages is more challenging than in emulsion-type sausages because solid-like materials should be created to replace solid fatty tissue (e.g., pork backfat). Amorphous cellulose, an insoluble fibre obtained from cereals, can form a gel, has no flavor or caloric value [119], and therefore can potentially be used in low-fat fermented sausages. Campagnol et al. [119] used amorphous cellulose gel to replace 25% to 100% of pork backfat in fermented sausages made of 85% meat and 15% pork backfat. As expected, fat and cholesterol contents were progressively and significantly lower with the increase in amorphous cellulose gel content (and decrease of pork backfat content). Moreover, the authors reported a significant content reduction of volatile compounds originating from lipid oxidation (saturated aliphatic aldehydes, unsaturated aliphatic aldehydes, ketones, and alcohols), which are important for the aromatic properties of fermented sausages. However, aroma-modified treatments received higher scores compared to the control, especially the treatment with total fat replacement. The authors also noticed that this treatment had a significantly higher content of sulfur compounds, which have strong odour impacts and are correlated with the cured and mature aroma of fermented sausages. The main negative impact was a significant decrease in sensory texture scores for treatments with replacement levels higher than 50%. In the study by Glisisc et. al. [120], 16% of pork backfat was replaced with an inulin gelled suspension in fermented sausages made of 75% meat and 25% pork backfat. A significantly lower fat content was reported, while no significant differences were found in terms of cholesterol, saturated fatty acids (SFA), and polyunsaturated fatty acid (PUFA) contents. Springiness and chewiness were significantly lower in the modified sausages. However, the results of sensory analysis (performed by a trained panel) indicated no significant differences in all observed properties—appearance, cross-section appearance, colour, odour and taste, texture, and overall acceptance. Similar to Campagnol et al. [119], the authors concluded that inulin gelled suspension can be used to create fermented sausages with improved functional properties.

The application of DFs in emulsion-type sausages is simpler—fibres can be added directly during grinding and mixing (during meat emulsion preparation) without pre-treatment. Henning et al. [115] replaced about 1/3 of pork backfat in beef sausages with 1% of three different types of pineapple DFs (different by particle size) and water (according to their water-binding properties). As expected, fat content was lower in the modified treatments. All modified treatments were lighter, less red, and more yellow compared to the control, and generally had lower values for hardness, chewiness, and springiness. The authors pointed out that the treatment containing DFs with the largest particle size (100–400 μm) was closest to the control regarding emulsion stability properties, cooking loss, and purge loss. They also emphasised that sensory analysis should be performed in order to determine whether instrumentally observed differences would be perceived negatively. In the research by Kurćubić et al. [121], 25% to 100% of pork backfat was replaced with cellulose fibre pre-hydrated in a 1:9 ratio. As in the previous studies, fat content and energy value were progressively and significantly lower in modified treatments. On the other hand, progressively and significantly higher content of total ω-6 FA was determined in modified treatments, which led to more unfavourable ω-6/ω-3 ratios. However, PUFA/SFA ratios were more favourable when 50% or more of the pork backfat was replaced. Instrumental colour parameters were similar in all modified treatments compared to the control, while treatment with 100% of replaced pork backfat had significantly lower hardness and chewiness compared to the control and other modified treatments. The panelists also noticed this, giving significantly lower grades for sensory texture, juiciness, and overall acceptability.

Regarding burger-type (patty-type) meat products, Polizer-Rocha et al. [122] used 1% pea fibre, characterised for insoluble fractions (with water in a 1:6 ratio), to replace around 39% (7% in formulation) of pork backfat. They reported no significant differences (compared to the control) in terms of pH, technological properties (cooking loss and size reduction), instrumental colour and texture analysis, and sensory analysis. On the other hand, replacement led to significant fat content reduction.

Previously cited research successfully created meat products with reduced fat (and energy value), with similar technological and sensory properties. However, these meat products did not have favourable fatty acid content. This could be overcome by the addition of oils with a favourable fatty acid profile, which should pretreated to increase oxidative stability. DFs can be used as stabilization agents due to their emulsification properties.

Glisic et al. [120] also used an inulin oil emulsion (with linseed oil) to replace 16% of pork backfat in fermented sausages made of 75% meat and 25% pork backfat. Significantly lower fat content was reported (compared to the control), as well as no significant differences in cholesterol content. However, unlike the treatment with inulin gel suspension (without oil), a significantly higher ω-3 FA and a more favourable ω-6/ω-3 ratio were observed. On the other hand, significantly higher (compared to the control) instrumental yellowness, lower hardness and chewiness, and increased oxidation susceptibility after storage were found. Therefore, the authors suggest further studies to overcome these observed pronounced alterations. Stajić et al. [116] used corn fibres for the pre-treatment of linseed oil (fibre:oil:water = 1:7:14) to replace animal fat in all-chicken frankfurters, in order to obtain products that can provide 50 and 100% of recommended daily alpha-linolenic acid intake. Cooking loss, purges loss, and proximate composition were not changed in modified treatments (compared to the control). Higher values of instrumental yellowness were attributed to linseed oil characteristics. Higher values of hardness and chewiness were observed in modified treatments. As expected, lower total SFA and higher total PUFA values were obtained in modified treatments. In addition, significantly favourable PUFA/SFA and ω-6/ω-3 ratios were also observed in modified treatments. The results of the sensory analysis showed that the modified treatments were similar to the control regarding colour, taste, odour, and overall acceptance.

The mentioned research undoubtedly pointed out that DFs can be used in the creation of low-fat meat products and/or those with improved FA profiles. However, the amount of fat replaced, the content of insoluble/soluble fibres, particle size, and oil properties should be considered.

As mentioned, DFs have the potential to be used in emulsion-type sausages as phosphate replacers. Phosphates promote myofibrillar protein solubilization and, therefore, are important for the formulation and stability of meat emulsions [127]. Since phosphates exhibit a synergistic effect with salt, they are of great importance in emulsion-type meat products with reduced salt content—research recommends the use of phosphates or their replacement in these products when salt content is reduced below 1.5–1.7% [44,45]. In the research by Powell et al. [118], replacement of 0.38% sodium tripolyphosphate (STPP) with 0.75% citrus fibre led to similar characteristics in low-salt Bologna-type sausages compared to the control, regarding total fluid separation, instrumental colour, and texture properties. On the other hand, higher fibre content led to higher hardness and chewiness values. No significant differences were found in terms of aroma, odour, off-flavour, and colour. The authors concluded that replacing phosphates with citrus fibre could not replace all STPP functional properties and that replacement must be product-dependent. Stajić et al. [117] used 0.3% and 0.6% of wheat, maize, pea, and potato fibres to replace 0.3% of phosphates in all-beef model system emulsion with reduced salt content (to 1.5%). The authors found significantly higher cooking loss in modified treatments, except in treatments with 0.6% of maize and pea fibres. Significantly higher hardness and chewiness, and lower springiness, were found in all modified treatments. Similar to the findings of Stajić et al. [117] and Powell et al. [118], Magalhães et al. [128] also reported significantly higher cooking loss in low-salt (1.5%) Bologna sausages when 0.5% of STPP was replaced with 2.5% bamboo fibre. However, an increment to 5% of DFs led to a similar cooking loss compared to the control. Additionally, the replacement of STPP with bamboo fibre led to significantly higher values of hardness and chewiness, and lower values of springiness. Although modified treatments were accepted by consumers, they had lower scores compared to the control.

As was pointed out in the cited research, the use of DFs as phosphate replacements has certain limitations. On the other hand, the use of other natural ingredients, such as natural calcium powders [128], also gives promising results (with certain limitations), as well as the introduction of novel processing techniques, such as the application of ultrasound [129]. Therefore, all of this offers great potential for a multi-strategic phosphate replacement approach.

## 5. The Importance of the Industrial Production of PBMAs and the Role of DFs in Their Enrichment/Fortification

The classification (a didactic division of meat analogues according to the origin of the source of added proteins instead of animal) includes the following: (1) plant proteins from soy, peas, gluten, etc.); (2) cellular proteins from cultured meat; (3) proteins obtained by fermentation—a protein-rich food ingredient derived from cultivated fungal mycelium; (4) proteins obtained from insects; and (5) proteins obtained from microalgae [130,131,132,133,134].

The above-mentioned PBMAs have a growing popularity among consumers and show an upward trend in the market. Meat analogues have inherent advantages, including a lower environmental impact/footprint (land, water, and carbon), the potential to supply a growing population with high-quality protein, and the absence of animal welfare and ethical issues when compared to conventional meat products. The food industry is focused on developing innovative PBMAs that can match the physical, functional, and sensory properties of conventional meat products. To create such products, certain advanced plant-based protein processing technologies are applied to improve texturization and functionality: electrospinning, extrusion, high-pressure processing, sonication, anti-solvent deposition, 3D/4D printing, mechanical elongation, shear structuring, mechanical elongation, and freeze-thaw alignment. The application of unconventional and sustainable plant protein sources could further improve sustainability in the future. The challenges that the meat analogue industry must address include the following: high processing levels, a deficit of bioactive compounds, and sensory properties of the analogues [135].

The growing demand for PBMAs is a result of the need to address environmental, health, and ethical issues. Mimicking the sensory properties of animal meat remains an insurmountable challenge for the PBMA industry. To achieve longer-term consumer acceptance of innovative PBMAs and foster a more sustainable food system, additional challenges must be addressed: production costs, appearance, texture, flavor, and nutritional value of the analogues. The potential for more efficient and environmentally friendly control of the fermentation process by judicious selection of starter cultures of microorganisms (bacteria and fungi) using modern methods has been investigated and established. The design of the technology for the production of starter cultures in the fermentation of plant materials, whose application is expected in the production of PBMAs, has not attracted sufficient attention, although it may contribute to better sensory quality. Favourable results are expected from the application of artificial intelligence and mathematical modeling in the creation of effective starter cultures of microorganisms by optimizing the fermentation they produce on plant material [136,137]. Therefore, each challenge must have its own solution pathways and key stakeholders who will create innovative products, such as PBMAs, by applying methods that are the result of scientific research [138].

Burgers were the top-selling PBMAs in 2019 (US$283 million), followed by sausages and hot dogs (US$159 million), and hamburgers (US$120 million). The predominant geographic presence of PBMA manufacturing companies (61) is in North America, with 17 located in Europe, and only a few in Asia, Australia, or Africa. There is also evidence that meat sales decreased by 5% from 2015 to 2019 in the US [133]. Data from many vegan societies and consulting companies report that people whose diets are based primarily on plant-based foods are increasing in number [139]. The global PBMA market is projected to grow from $1.89 billion in 2021 to $4.04 billion by 2027 [140]. The conclusion is that it is necessary to continuously work on additional innovations in order to create healthy and safe meat substitutes [141].

For a long time, great attention has been paid to the addition of plant-based proteins to meat products, but today, it has become clear that dietary fibre (DF) can make an immeasurable contribution to the physicochemical, textural and other properties of traditional processed meat products and PBMAs. DF favorably affects a number of extremely important techno-functional properties of PBMAs, such as gel strength, emulsion stability, WHC, microstructure (uniform, porous), and texture (hardness, elasticity, cohesiveness, and chewiness). In order to achieve the desired results, the correct choice of the DF source (interactions with other components, chemical structure, solubility, size, concentration, and processing conditions) must be made [81]. Today, there is data available on many commercial PBMAs that use various added DFs. From a nutritional perspective, the amount of DFs in PBMAs is of great importance to consumers. Products that contain at least 3 g of fibre per 100 g of product can be labeled as a “source of fibre”; a product “rich in fibre” must contain at least 6 g of fibre per 100 g of product (data for fortified products in EU countries) [142].

Technological parameter tests in PBMA samples to which DFs were added determined enhanced WHC, reduced cooking losses, and increased production yield. The addition of DFs has a positive impact on the quality of products with low-fat content because it stabilizes the emulsion and improves their texture. Chemical tests reveal increased content of DFs and minerals and reduced fat content. Optimization of the type and amount of added fibres is necessary to obtain meat products with improved properties [143]. The integration of DFs into meat product formulations contributes to improving functional and sensory properties and enriching nutritional value and needs in order to meet consumer demands and desires [59].

The innovation of meat products enriched with plant-based ingredients (as the main sources of DFs) is achieved by their inclusion as an essential element in the diet, in order for the human body to function properly. In processed meat products, such fortification contributes to the nutritional improvement of these increasingly sought-after products (current trends in the consumption of mainly animal products, in a diet rich in proteins). In general, the incorporation of DFs has been mainly attempted in meat products with a minced structure (salami, sausages, burgers, meatballs, emulsified products with a higher degree of grinding) or restructured products (meatballs, restructured meat slices, rolls). The application of different types of DFs can be optimised when it comes to physicochemical, sensory, and nutritional parameters. DFs from different plant sources exhibit different functional properties (gelling ability, solubility, viscosity, WHC, and oil absorption) that directly affect the properties of the final product [143].

These effects better maintain juiciness (less losses during thermal processing, preservation of aroma and taste). DFs can reduce fat content, improve texture and juiciness, and modify taste, aroma, and color. Enriched products can have a lower energy value due to reduced fat content, which promotes a healthy and balanced diet. Through optimization, products with an improved nutritional profile and high sensory acceptability can be obtained [143].

The production of meat products enriched with fibre from plant sources is an innovative solution that combines several advantages related to improving nutritional value, regulating fibre intake, meeting the demands of consumers who emphasise a healthy lifestyle, as well as hybrid foods specific to busy lifestyles. Future research horizons include investigating the potential of meat products enriched with DFs for long-term health effects, particularly in improving cardiovascular and gastrointestinal health during prolonged continuous consumption [143].

## 6. Conclusions and Future Perspectives

The incorporation of DFs into meat and meat analogue products offers significant nutritional, techno-functional, and environmental benefits. Fibres contribute to reducing the fat and caloric content of meat products, making them more appealing to health-conscious consumers. For instance, soluble fibres like oat β-glucan and resistant starch have been shown to lower fat absorption and improve the texture of low-fat meat formulations, while also enhancing satiety. Additionally, fibres derived from plant sources such as grape pomace, peas, and oats exhibit prebiotic properties that promote gut health by supporting the growth of beneficial microbiota like Bifidobacteria and Lactobacilli [15]. These fibres also possess antioxidant properties that can mitigate the formation of process-induced toxicants, such as advanced glycation end-products (AGEs) and polycyclic aromatic hydrocarbons (PAHs), thereby enhancing the safety and nutritional profile of meat products [65,85].

From a technological perspective, fibres play a critical role in PMA production by influencing texture, emulsification, moisture retention, and shelf life. For example, pea and soy fibres have been shown to improve the fibrous texture and structural integrity of plant-based meat alternatives, while seaweed-derived fibres like alginate and carrageenan enhance water retention and emulsion stability [18,83,]. However, incorporating fibres can present challenges, particularly in optimizing texture and maintaining sensory characteristics such as flavour and colour. Strategies such as adjusting processing techniques, combining fibres with other functional ingredients, and using advanced technologies like high-pressure homogenization have shown promise in overcoming these challenges, resulting in fibre-enriched products with improved stability and consumer appeal [144,145].

With the growing demand for healthier and more sustainable meat alternatives, emerging technologies offer new opportunities to further enhance the fibre’s functional properties. Advanced processing techniques, such as extrusion, high-pressure homogenization, and fermentation, provide innovative ways to incorporate fibres without compromising texture or product quality [96,144]. For instance, fermentation has been shown to improve the bioavailability of nutrients in fibres, while extrusion can enhance their textural properties in PMAs [98]. Additionally, microencapsulation techniques can be used to enhance the health benefits of fibres by improving the controlled release and bioavailability of their bioactive compounds [97].

The use of fibres derived from agricultural byproducts, such as fruit and vegetable peels, can contribute to sustainability by reducing food waste but also offer functional benefits, such as improved water retention and texture enhancement in meat analogues [11,79]. For example, apple and citrus fibres have been successfully used to enhance the texture and juiciness of low-fat meat products while also providing antioxidant benefits [93,94]. Furthermore, the development of hybrid meat products, which combine animal and plant-based ingredients, opens up new opportunities for fibre incorporation. These hybrid products can leverage the functional properties of fibres to improve product quality while meeting consumer demand for sustainable and healthier alternatives [19].

In conclusion, DFs are versatile ingredients in the development of healthier and more sustainable meat and meat analogue products. By addressing current challenges and leveraging emerging technologies, the food industry can continue to innovate and meet the growing demand for functional, environmentally friendly food options. Future research should focus on exploring novel fibre sources and evaluating the long-term health benefits of fibre-enriched meat and PMA products to ensure their success in the global market.

## Figures and Tables

**Table 2:** Influence of the use of fibres in fat-reduced and/or fat-improved meat products.

Meat Product	Fibre/Amounts in Formulation	% of Oil in Formulation *	% Fat Reduction in Product	PUFA/SFA	$\frac{\omega 6}{\omega 3}$	Reference
Fermented sausages	Amorphous cellulose gel/3.75–15%	0	33.9–75.8	nd	nd	[119]
Dry-fermented sausage	Inulin gelled suspension/16%	0	29.3 *	0.34 *	13.22	[120]
Beef emulsion-type sausage	Pineapple dietary fibres/10%	0	≈45 *	nd	nd	[115]
Pork emulsion-type sausage	Cellulose fibre gel/5–20%	0	22.5–70.2 *	0.52–0.64	21.95–34.26	[121]
Emulsion-type model system	Amorphous cellulose fibre/0.2–1.5%	0	11.2–70.8	nd	nd	[114]
Beef burger	Pea fibre/1% fibre +6% water	0	18.7	nd	nd	[122]
Dry-fermented sausage	Inulin gelled emulsion (linseed oil)/16%	3.2 *	20.3	0.58 *	2.23	[120]
Emulsion type (chicken frankfurter)	Corn fibre emulsion (linseed oil)/6.25% and 12.5%	2–4	0	0.96–1.38	0.41–0.67	[117]

* Approximated values calculated from research data (%); nd—not determined.

## Data Availability

No new data were created or analysed in this study. Data sharing is not applicable to this article.
